# Activated Carbon Derived from Carbonization of Kevlar Waste Materials: A Novel Single Stage Method

**DOI:** 10.3390/ma14216433

**Published:** 2021-10-27

**Authors:** Daniel Karthik, Vijay Baheti, Jiri Militky, Muhammad Salman Naeem, Veronika Tunakova, Azam Ali

**Affiliations:** 1 Department of Materials Engineering, Faculty of Textiles, Technical University of Liberec, 46117 Liberec, Czech Republic; jiri.militky@tul.cz (J.M.); veronika.tunakova@tul.cz (V.T.); azam.ali@tul.cz (A.A.); 2 Department of Textile Technology, Indian Institute of Technology, Delhi 110016, India; vijaykumar.baheti@gmail.com; 3 Faculty of Engineering and Technology, National Textile University, Faisalabad 37610, Punjab, Pakistan; salman.ntu@gmail.com

**Keywords:** activated carbon, carbonization, porous surface, cost effective, Kevlar, textile waste, recycle, electrical conductivity, EMI shielding

## Abstract

The augmented demands of textile materials over time have brought challenges in the disposal of substantial volumes of waste generated during the processing and end of life of such materials. Taking into consideration environmental safety due to discarding of textile waste, it becomes critical to recuperate useful products from such waste for economic reasons. The present work deals with the preparation of porous and electrically conductive activated carbon fabric by a novel single stage method of simultaneous carbonization and physical activation of Kevlar feedstock material procured from local industries, for effective electromagnetic (EM) shielding applications. The Kevlar fabric waste was directly carbonized under a layer of charcoal without any intermediate stabilization step at 800 °C, 1000 °C, and 1200 °C, with a heating rate of 300 °C/h and without any holding time. The physical and morphological properties of the activated carbon, influenced by carbonization process parameters, were characterized from EDX, X-ray diffraction, SEM analysis, and BET analysis. Furthermore, the electrical conductivity was analyzed. Finally, the potential application of the activated material for EM shielding effectiveness was analyzed at low (below 1.5 GHz) and high (2.45 GHz) frequencies. The phenomena of multiple internal reflections and absorption of electromagnetic radiations was found dominant in the case of activated carbon fabric produced at higher carbonization temperatures.

## 1. Introduction

The ever-pressing affairs on global warming and ecological complications persistently impact the rising awareness in societies of the importance of sustainability, circular economy, and recyclable/reusable products. [1,2,3]. With the increasing demand for textiles, the amount of waste generated during the processing and end of life of textile materials has significantly increased, adding to the challenges encountered due to its disposal thereof. Tons of textile waste is produced annually from textile industries. The textile waste generated by European Union (EU) countries equates to approximately 16.5 million tons annually [4]. A vast amount of post-consumer textile waste is thrown into landfills or is incinerated and left to decompose, usually releasing methane, among other toxic fumes, which contributes in the greenhouse effect of global warming. Textile wastes from specific categories of materials, that are expensive and divergent, retains its characteristic properties and, due to economic and ecological reasons, it is essential to pave the way to potentially reuse/recycle the same [5,6,7]. The typical high value-added products obtained from such wastes are nanoparticles, nanocomposites activated carbon, and fillers for reinforcements, to mention a few.

Activated carbon is a highly porous product, usually derived from semi crystalline precursors such as viscose rayon, phenolic resins, polyacrylonitrile, or isotropic coal tar pitches, by a number of lengthy and multistep processes [8,9,10]. Activated carbon has a very high porous structure with a large internal surface area of around 500–2000 m^2^/g [11]. In recent years, research on exploring alternative, inexpensive sources together with methods for the preparation of activated carbon materials has attracted attention. Activated carbon is an excellent adsorbent for the removal of dyes, heavy metals, hazardous smoke, unnecessary odor, taste, and organic substances from the environment [12]. The large surface area, well-developed internal structure, and presence of various surface functional groups depend on the nature of the raw material used, the nature of the activating agent, and the conditions of the pyrolysis and activation processes [9,13].

Surprisingly, the utilization of highly ordered polymer precursors (i.e., Aramid fibers) which do not need oxidative stabilization, have been studied for activated carbon fiber production [14]. Aramid is a long chain aromatic polyamide where at least 85% of amide linkages are attached directly to two aromatic rings [8]. Freeman et al. [14] first proposed the use of Kevlar to obtain carbons with high surface areas, narrow pore size distributions, and acceptable yields [9,15]. Later, a number of studies focused on the preparation of activated carbon fibers from Nomex and Kevlar fibers by physical activation using CO_2_ or steam, and by chemical activation in the presence of small amounts of phosphoric acid [16,17,18,19,20]. More recently, the introduction of intermediate isothermal treatments during the pyrolysis of Aramid fibers have been reported to improve the carbon yield, nitrogen content, and reversible electrical capacity of activated carbon fibers [15,21,22]. Despite all these previous efforts, Aramid fibers are still not attractive precursors of carbon materials due to their sophisticated applications in the aeronautical and aerospace industries. An alternative option of using Kevlar pulp has been employed previously as inexpensive feedstock, but surprisingly there are no studies on carbonization of Aramid fibrous textile waste [23]. 

For general and more commonly used textile fibers, there are plenty of possibilities and various processes to recycle/reuse fibrous wastes. The approaches include chemical processes to depolymerize polymers, recovery of plastic resins from carpet fibers, direct extrusion of mixed fiber waste, composites as wood substitutes, fibers for concrete and soil reinforcement, waste-to-energy conversion, and carpet as feedstock for cement kilns, to mention a few. However, for high performance fibers, such as Kevlar in particular, it is relatively harder to maintain its original structure during processes intended for reutilization of fibrous wastes. 

Numerous efforts have been made in previous studies on surface modifications using chemical etching by mixed acids, ionization, surface irradiation by plasma, ultrasonic treatment, chemical grafting methods, and fluorinated modification [21,22]. However, the decomposition mechanism of Aramid has relatively fewer studies for the development of porosity or surface activation behavior to create micro porous Aramid structures using controlled thermal treatment and carbonization.

**Figure 1:**
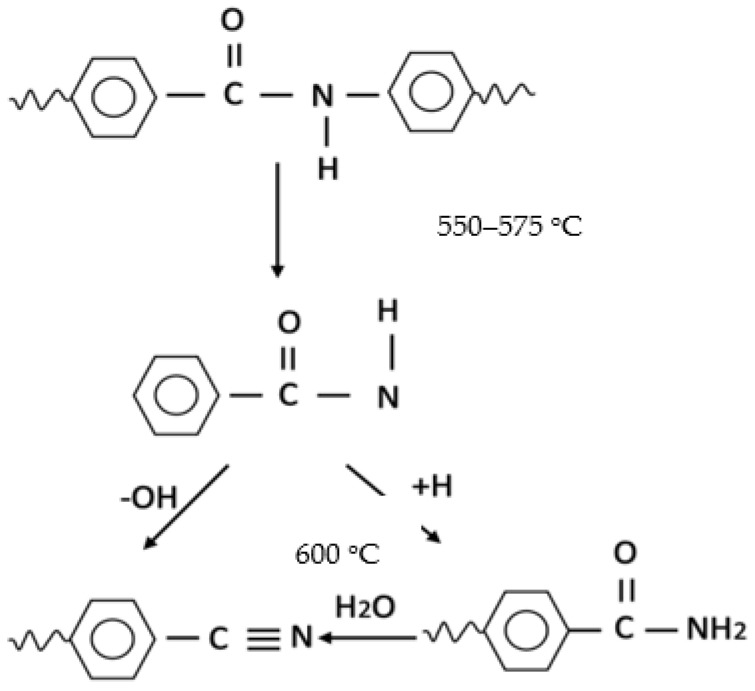
Representation of progressive decomposition process of the Kevlar structure [26].

In our previous study, Naeem et al. [24], a single stage carbonization and physical activation method, under a layer a charcoal, was employed to produce a porous and electrically conductive activated carbon web from needle punched acrylic fibrous waste, but with the necessity of an additional step of thermal stabilization, wherein dehydrogenation, oxidation, and nitrile cyclization reactions take place prior to subsequent carbonization. The present work focuses on the preparation of porous and electrically conductive activated carbon fabric from Kevlar fabric waste, using the same single stage carbonization and physical activation process, except in this case there is no necessity for a thermal stabilization step. Due to rigid-rod structure and a high degree of aromaticity of Kevlar, the direct carbonization process is used. As described in Figure 1, during the carbonization process at a temperature of about 550–575 °C, the progressive decomposition of amide groups take place, wherein intermediate aryl nitrile species are formed. Above 600 °C, polyaromatic compounds are created. This is a continuous single stage process, without the need for an additional thermal stabilization step [25,26].

The carbonization process employed is a less expensive treatment with a lower production time and cost, due to pyrolysis conditions of direct carbonization without intermediate stabilization, in turn leading to less energy consumption, which means less waste. Charcoal, being inexpensive and readily available, was used as the source of heat and to facilitate pyrolysis, which adds to a cheaper production cost. 

The positive approach to utilizing textile waste materials in seamless amalgamation with the distinct properties of Kevlar, the novel and faster single stage carbonization process, and the inexpensive and easily obtainable charcoal for purpose of incineration has directed us to the notion of possibly developing a tecno-economic product with potential applications. The novelty of present work was the utilization of natural gas for the simultaneous development of porous and electrically conductive Kevlar fibers using controlled physical activation and carbonization. The activated carbon fabrics produced were tested on a waveguide method and a coaxial transmission line method for their end use performance as effective electromagnetic shielding materials. In this way, the large quantity of Kevlar fibrous waste can be utilized for advanced applications to protect the nature.

## 2. Experimental Methods

### 2.1. Materials

The Kevlar woven fabric waste was procured from Veba Textile Mills Ltd (Broumov), Czech Republic as short, discarded fabric waste. The Kevlar had a 1710 dtex yarn count, 0.24 m^2^/g BET surface area, 1.43 g/cm^3^ density and 5.2 wt% moisture content. Its elemental analysis was reported as weight percentages on a dry basis of 63.85% C, 15.09% N, 0.8% S, and 18.84% O.

### 2.2. Preparation of Activated Carbon Fabric

**Figure 2:**
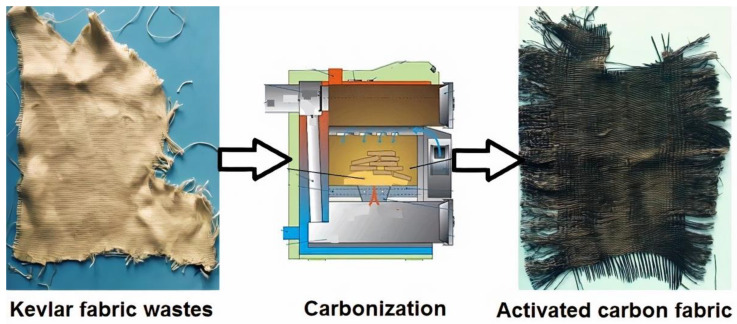
Representation of activated carbon fabric preparation from Kevlar fabric waste.

The novee single stage carbonization and physical activation of Kevlar fibrous waste was carried out under a layer of charcoal in a high temperature furnace (Elektrické Pece Svoboda, Svetice, Czech Republic). The Kevlar fabric was directly carbonized without any intermediate stabilization step. Temperatures of 800 °C, 1000 °C, and 1200 °C at 300 °C/h heating rate and with no holding time were adopted during the process of carbonization. Figure 2 shows the different stages of production of activated carbon fabric from Kevlar fibrous waste.

### 2.3. Characterization of Activated Carbon Fabric

***Characterization of physical properties.*** The change in yarn count, yarn strength, fabric areal density, thickness, shrinkage, flexibility, and dusting tendency of Kevlar was studied with respect to the increase in the carbonization temperature. The single yarn strength was measured on a TIRA test 2300 LaborTech machine (LaborTech, Opava, Czech Republic) with a gauge length of 20 cm and at a speed of 100 mm/min. The ASTM D 2259 standard [27] was employed for the shrinkage estimation, where change in length of the Kevlar yarn before and after carbonization was measured. For the change in flexibility after carbonization, the bending length was measured by employing the principle of a cantilever bending under its own weight as per ASTM D 1388 standard [28]. The TH-7 instrument (Faculty of Textiles, Technical University of Liberec, Liberec, Czech Republic) was used to measure the bending force of samples and then stiffness was calculated [29]. Lastly, Taber wear and abrasion testers (Taber Industries, New York, NY, USA) were used as per ASTM D 3884 standard [30] to rub the surface of the carbonized Kevlar fabric under 30 rotating cycles, and the amount of generated dust particles were used to evaluate the dusting tendency. 

***Energy dispersive X-ray (EDX) analysis.*** An LZ 5 EDX detector, Oxford Instruments, Oxford, UK was used to calculate the relative percentage of different elements in the activated material at three selected carbonization temperatures. 

***X-ray diffraction (XRD) analysis.*** The XRD analysis was carried out to confirm the development of crystalline and amorphous regions in prepared activated carbon fabric with respect to the change in carbonization temperature. It was performed on a PANalytical X’ Pert PRO MPD diffraction system. 

***SEM analysis.*** The porosity characteristics in activated carbon fabric were observed under the field emission scanning electron microscope Sigma, Zeiss, Germany. The 2-kV accelerated voltage and 1000× magnification was employed to obtain the surface micrographs of activated carbon fabrics produced at 800 °C, 1000 °C, and 1200 °C carbonization temperatures. The activated carbon fabric obtained was electrically conductive, so it’s metallization was not needed before testing. Nevertheless, the received Kevlar fabric was metalized by sputter coating.

***BET surface area.*** An Autosorb iQ, Quantachrome Instruments, Boynton Beach, CA, USA was employed to measure the specific surface area of carbonized Kevlar fabrics from N_2_ adsorption–desorption isotherms at 77.35 K. The relative pressure range P/P_0_ from 0.02 to 1 was adopted for the collection of adsorption/desorption isotherm measurements. Before the adsorption analysis, the produced activated carbon samples were pre-treated in an oven at 45 °C for 5 h and then out gassed overnight at 300 °C. Lastly, the specific surface area was determined from the calculations of obtained adsorption and desorption isotherms.

***Characterization of electrical resistivity.*** The surface and volume resistivity of activated carbon fabric prepared at 800 °C, 1000 °C, and 1200 °C was determined by using a Hewlett Packard 4339 B resistance meter at a relative humidity of 40%, at a temperature of 22 °C, and at 100 V according to ASTM D257-14 [31]. The direct current was applied at a voltage of 100 ± 5 V at opposite ends of the activated carbon fabric and the resultant current passing through the sample was measured after 15 ± 1 s.

### 2.4. Electromagnetic Shielding Effectiveness of Activated Carbon Fabric

The two different measurement principles were employed to estimate the electromagnetic shielding effectiveness of prepared activated carbon fabric. The waveguide method was used at a higher frequency (i.e., 2.45 GHz), whereas the coaxial transmission line method for a lower frequency range (i.e., 600 MHz to 1.5 GHz) was used.

***Waveguide method.*** A receiving antenna was placed inside of a rectangular hollow waveguide having electrically conductive walls, whereas a sample was placed at the entrance to the waveguide. The electromagnetic waves were generated by a network analyzer Agilent E 4991A. A high frequency analyzer HF-38B (Gigahertz Solutions, Langenzenn, Germany) was used to receive the electromagnetic signals. The complete details of this measurement method can be found in previous work [32]. The Equation (1) was used to estimate the electromagnetic shielding effectiveness, *SE* [dB]

(1) $$SE=10\;\log\frac{P_{t}}{P_{i}}$$

where *P_t_* and *P_i_* is power density (W/m^2^) measured in presence of sample (transmitted), and without the sample (incident,) respectively. 

***Coaxial transmission line method.*** The insertion-loss principle, according to ASTM D 4935-10 standard, was employed to determine the EM shielding effectiveness in this method. The measurement set-up consisted of a sample holder with its input and output connected to the network analyzer. A shielding effectiveness test fixture (Electro-Metrics, Inc., model EM-2107A, Johnstown, NY, USA) was used to hold the sample. The network analyzer (Rohde & Schwarz ZN3, Rohde & Schwarz, Columbia, MD, USA) was used to generate and receive the electromagnetic signals. 

## 3. Results and Discussions

### 3.1. Physical Properties of Activated Carbon Fabric

Table 1 shows the effect of carbonization temperature on physical characteristics of Kevlar derived activated carbon fabrics. The yarn count, areal density, and thickness of Kevlar fabrics were found to drop by more than 50% after the carbonization at 1200 °C. During the carbonization, the main chemical transformation occurred, involving the formation of intermediate aryl nitriles at 500–600 °C, and then progressive aromatization/ring condensation, leading to polyaromatic compounds above 600 °C. This resulted in isotropic, non-graphitizable carbon fibers at first and then into activated carbon by activation with oxidizing natural gas [33]. The yield of activated carbon reduced with an increase in carbonization temperature, and 31% yield was obtained after 1200 °C carbonization. These results are in agreement with previous studies [15,22].

**Figure 3:**
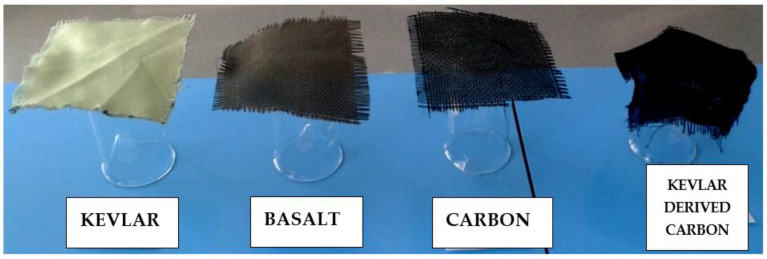
Flexibility of produced activated carbon fabric and other regular fabrics.

When shrinkage testing was carried out, the Kevlar fabrics showed a negligible change in length upon carbonization, unlike our previous experience on acrylic fibers [24]. This indicated better thermal stability of Kevlar fabrics due to the highly ordered arrangement of macromolecules. Nevertheless, the dusting tendency of Kevlar was observed to be similar to the case of acrylic fibers with an increase in carbonization temperature. Interestingly, the flexibility of carbonized Kevlar was found to be superior to the regular carbon and Kevlar fabric (See Figure 3). This indicated better drape properties of the produced activated carbon fabrics and, thus, increased comfort when used as personal protective fabrics.

To know the draping and the physical comfort of Kevlar derived activated carbon fabrics, the stiffness was measured from the bending properties of the fabric using bending length and flexural rigidity. As shown in Table 2, the stiffness was found to reduce with an increase in carbonization temperature. This behavior was attributed to increased porosity, reduced inter-fiber/yarn friction, and abrasion at fiber/yarn cross-over points due to the decomposition of organic substances from Kevlar fabric during the carbonization. 

**Figure 4:**
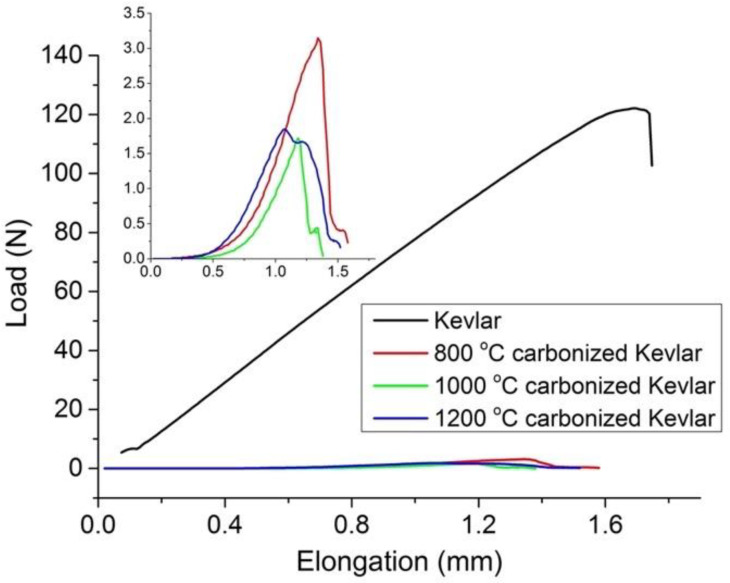
Load elongation curve for carbonized Kevlar fabrics.

**Figure 5:**
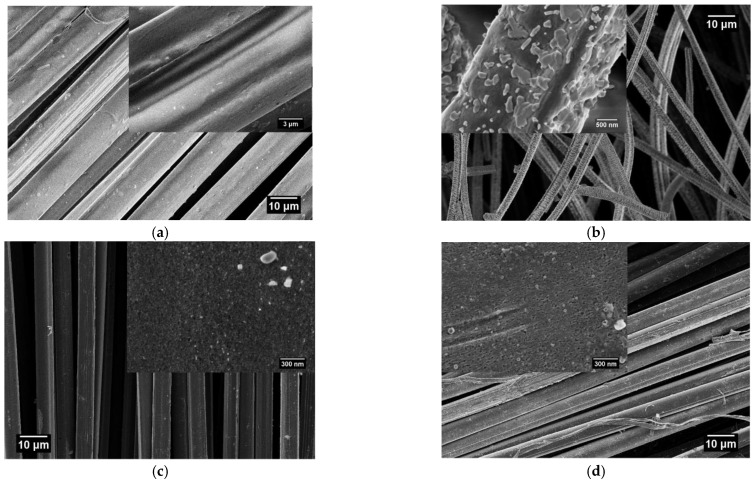
Microstructure of Kevlar fibers after different carbonization temperatures. (**a**) Kevlar fabric; (**b**) activated carbon fabric at 800 °C; (**c**) activated carbon fabric at 1000 °C; and (**d**) activated carbon fabric at 1200 °C.

Finally, when mechanical properties were investigated, the single yarn strength of Kevlar was found to reduce heavily after the carbonization (Figure 4). The maximum reduction in yarn strength was observed in the case of 1200 °C carbonized Kevlar fabric, where breaking force decreased from 127 N to 2 N and breaking elongation decreased from 1.79% to 0.94%. This behavior was attributed to the formation of additional pores or rough surfaces on Kevlar fibers after the carbonization (see Figure 5). 

### 3.2. Characterization of Activated Carbon Fabric 

**Figure 6:**
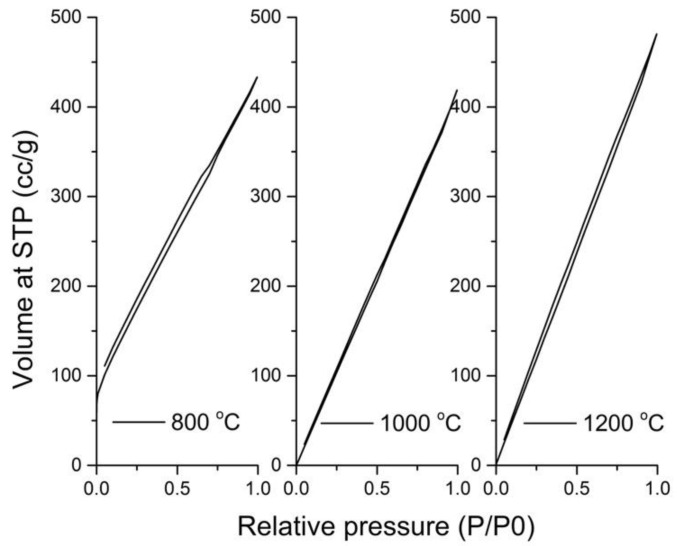
Nitrogen adsorption-desorption isotherm of carbonized Kevlar fabrics.

***BET analysis.*** The absorption of electromagnetic radiations depends on the specific surface area of materials; therefore, the BET analysis was performed on samples of Kevlar derived carbon obtained at 800 °C, 1000 °C, and 1200 °C carbonization temperatures. From the nitrogen adsorption/desorption isotherm of activated carbon fabrics shown in Figure 6, a rapid rise in the adsorption–desorption isotherm can be observed. This confirmed the type I isotherm based on the classification of the International Union of Pure and Applied Chemistry (IUPAC) [34,35] and the formation of micropore in carbonized Kevlar fabrics. Moreover, the constant slope at intermediate relative pressures with increasing carbonization temperatures indicated the presence of uniform micropore sizes [13]. More clear observation of the isotherm indicated some contribution of type IV with a type H4 hysteresis loop. With an increase in carbonization temperature, the type H4 hysteresis loop was found to become narrow. This indicated a negligible effect of an increase in carbonization temperature on the development of mesoporosity in the structure [36,37]. The marginal increase in specific surface area with an increase in carbonization temperature was observed. The specific surface area for activated carbon fabric prepared at 1200 °C, 1000 °C, and 800 °C obtained was 248 m^2^/g, 174 m^2^/g, and 109 m^2^/g, respectively. This behavior was attributed to the opening of previously inaccessible pores through the removal of tars and disorganized carbon by the gradual reaction of atmospheric oxygen with carbonized Kevlar fibrous waste [38]. Nevertheless, the smaller values of maximum surface areas compared to literature can be associated to possible collapse or closure of pores by a gasification-induced densification at higher carbonization temperature [24]. 

***Mechanism of pore formation.*** The porosity and surface area created during physical activation can be attributed to the loss of small molecules (CO and CO_2_) by gasification of non-graphitic carbon and heteroatoms, the reaction with graphitic carbon, and the reorganization of layers of pseudo-graphitic planes. The development of micro porosity in the present work can be related to selective gasification of the less ordered fractions of the carbonaceous material and an increase in the thickness of the layers of graphitic planes after the carbonization [37]. Moreover, the tendency to attain a limit in pore development with an increase in carbonization temperature can be explained in terms of the gasification-induced densification effect [12].

***SEM morphology.*** The formation of porosity in carbonized Kevlar fibrous waste was observed from the Kevlar fiber surface before and after carbonization. The SEM images of Kevlar fibers at different carbonization temperatures of 800 °C, 1000 °C, and 1200 °C can be seen in Figure 5a–d. The Kevlar fibers showed a prominent rough surface after the carbonization, and the surface roughness was increased with increasing carbonization temperature. Therefore, development of a more porous structure was confirmed post physical activation of Kevlar fibers during the carbonization process [14]. 

***EDX analysis.*** The relative proportion of different elements present in the activated carbon fibers was estimated from the EDX analysis. With an increase in carbonization temperature from 800 °C to 1200 °C, the carbon content was found to increase and the oxygen content was found to reduce (See Table 3). This indicated the decomposition of Kevlar macromolecules at a higher temperature and the subsequent removal of hydrogen, sulfur, nitrogen, and other elements [39]. The 89.26% carbon content and 5.87% oxygen content were estimated for the activated carbon obtained at 1200 °C.

**Figure 7:**
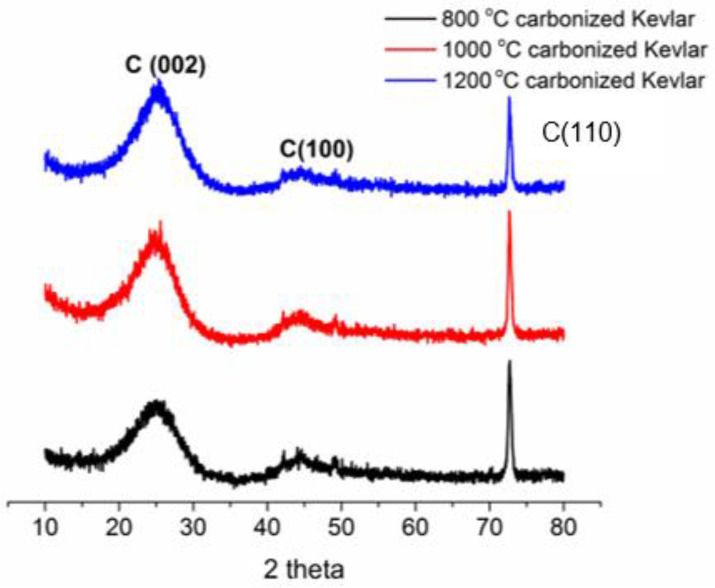
Effect of carbonization temperature on crystallinity of activated carbon fabric.

***XRD analysis.*** The XRD analysis was carried out to know the development of crystallinity with an increase in carbonization temperature. The nature of peaks observed in the XRD pattern can be used to identify the crystallinity of activated carbon samples produced at 800 °C, 1000 °C, and 1200 °C temperature (Figure 7). The development of a higher crystallinity at a higher carbonization temperature was confirmed based on the increase in intensity and sharpness of peaks with an increase in carbonization temperature. The presence of a hexagonal graphitic structure due to C (002) reflection was confirmed from the location of the strongest diffraction peak found at 25.5° [40]. The other diffraction peak found at 43° was associated with C (100) diffraction of the graphitic structure. Furthermore, more transformation of the amorphous structure into graphitized structure can be expected due to the reduced spread of XRD spectra with an increase in carbonization temperature. Therefore, higher electrical conductivity of activated carbon samples can be expected when they are produced at a higher carbonization temperature.

***Electrical conductivity.*** The electrical conductivity was measured to estimate the performance of EM shielding recorded by the reflection of EM radiations. The effect of carbonization temperature on electrical conductivity of activated carbon fabrics is shown in Table 4. With an increase in carbonization temperature, both surface and volume resistivity values were found to decrease. Almost 15,000- and 2500-times reduction in surface and volume resistivity, respectively, was observed for the 1200 °C activated carbon sample in comparison to the 800 °C activated carbon sample. The formation of more graphitization, confirmed by the existence of a sharp diffraction peak in XRD spectra (Figure 7), can be related to the higher electrical conductivity of the 1200 °C activated carbon sample.

**Figure 8:**
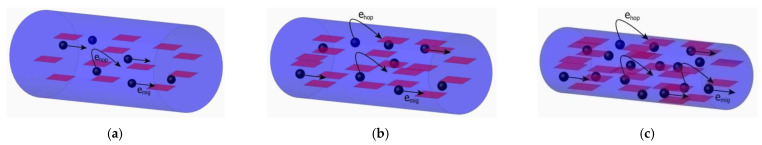
Mechanism of charge transport in activated carbon fabric [24] at (**a**) 800 °C, (**b**) 1000 °C, and (**c**) 1200 °C.

***Mechanism of charge transport.*** The movement of electrons through one graphite layer or their hopping across the defects/interfaces between disordered graphite layers can be attributed to the development of electrical conductivity in activated carbon samples [41,42,43]. Therefore, the two potential means of electron transport (migration and hopping) in the activated carbon fabric produced at 800, 1000, and 1200 °C, respectively are shown in Figure 8a–c. The migration and hopping of electrons were responsible for the higher electrical conductivity of the 1200 °C activated carbon fabric than the 800 and 1000 °C activated carbon. This was possible due to a higher graphite content, uniform distribution of graphite layers, and reduced fiber diameter, which finally resulted in the formation of a more compact micro-current network in the 1200 °C activated carbon fabric [44,45].

### 3.3. Electromagnetic Shielding Ability

**Figure 9:**
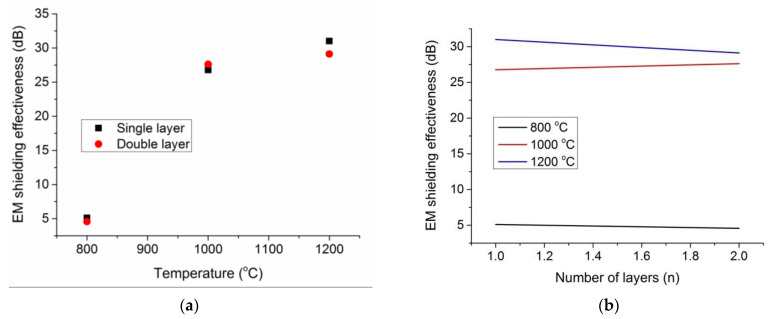
Electromagnetic shielding effectiveness at 2.45 GHz; (**a**) effect of carbonization temperature; and (**b**) effect of number of layers.

***Waveguide method.*** The electromagnetic shielding effectiveness of the prepared activated carbon fabric was measured in single and double layers at 2.45 GHz frequency using the waveguide method. When the carbonization temperature was increased, the Kevlar derived activated carbon exhibited an increase in the electromagnetic shielding effectiveness (see Figure 9). At the carbonization temperatures of 1200 °C, 1000 °C, and 800 °C, the EM shielding effectiveness of 31 dB, 27 dB, and 5 dB was found, respectively, for a single layer of activated carbon fabric. The shielding effectiveness was almost zero at a very low carbonization temperature, however, it increased drastically with the increase in carbonization temperature. For maximum shielding effectiveness, the location of the percolation threshold can be expected between 1000 °C and 1200 °C carbonization temperatures. In this temperature range, the carbonized Kevlar showed higher electrical conductivity, higher porosity, and higher surface area, and therefore increased multiple internal reflections and stronger absorption of electromagnetic radiations [46]. Nevertheless, the effect of increasing the number of layers was found to be negligible for increasing the shielding effectiveness (see Figure 9b). These results are not in agreement of our previous work on activated carbon web produced from acrylic fibrous waste. This behavior can be explained due simple interlacing of Kevlar warp and weft in woven fabrics, unlike more intermingled random arrangements of acrylic fibers in needle punched nonwoven webs of previous work [24].

**Figure 10:**
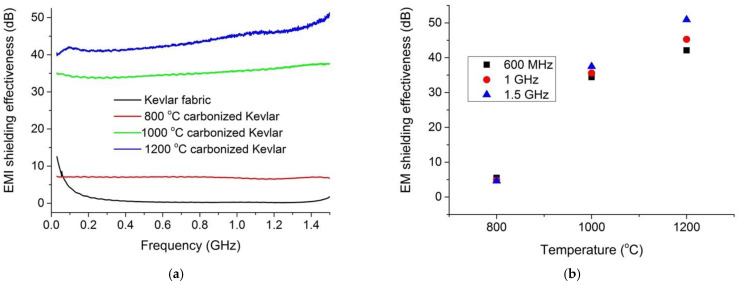
Electromagnetic shielding effectiveness in low frequency region; (**a**) effect of frequency; and (**b**) effect of carbonization temperature.

***Coaxial transmission line method.*** This method was employed to estimate the electromagnetic shielding effectiveness of activated carbon samples in frequencies of 600 MHz, 1 GHz, and 1.5 GHz. From Figure 10a,b, it can be seen that the shielding effectiveness increased with increase in the carbonization temperature. The lowest electromagnetic shielding effectiveness, of about 5 dB, was observed for the single layer of activated carbon fabrics produced at 800 °C. However, when the Kevlar fabric was carbonized at 1200 °C, the shielding ability of 42 dB, 45 dB, and 51 dB was found for respective frequencies of 600 MHz, 1 GHz, and 1.5 GHz. As discussed in the previous section, the similar reasons for the fiber arrangement and gasification-induced densification effect can be attributed to overall lower values of electromagnetic shielding for Kevlar derived activated carbon fabrics than the acrylic derived activated carbon nonwoven web in previous work [24].

***Mechanism of EM shielding.*** The EM shielding efficiency of materials is governed by the means of reflection, absorption, and multiple internal reflections of EM radiations. The cause of mismatch in impedance between air and material is due to the availability nomadic or mobile charge carriers (electrons or holes) on the surface result in the reflection of electromagnetic radiations [41,45]. The thickness of the material decides the second important mechanism of absorption of electromagnetic radiations caused by Ohmic loss and polarization loss [44]. The dissipation of energy by nomadic charges through conduction, hopping, and tunneling mechanisms results into the ohmic loss. Polarization is derived from functional groups, defects, and interfaces within the material, which leads to polarization loss from the energy required for overcoming the momentum to reorient the dipoles in each half cycle of the EM wave. Lastly, the inhomogeneity and huge interfacial area of materials contribute to the mechanism of multiple internal reflections due to the scattering effect within the shielding material [46]. 

In present work, the higher EM shielding properties of the 1200 °C activated carbon fabric can be attributed to the increased multiple internal reflection and stronger absorption of EM waves. This is because the carbonization of Kevlar at a higher carbonization temperature produced a heterogeneous surface with increased electrical conductivity and porosity. This can be explained further from the increased graphite content, uniform dispersion of graphite layers, reduced fiber diameter, etc., of the 1200 °C activated carbon fabric shown in Figure 4, Figure 6d and Figure 7. The dissipation of more electrical energy and, thus, higher Ohmic loss can be expected from the greater number of nomadic charges (from increased graphite content in Figure 8) coupled with their uniform state of dispersion for elongated electrons’ mean free paths and enhanced conductive network [47]. Further, the dissipation of the incident EM wave can be expected from the reduced fiber diameter (Table 1) and larger conductive surface area (Figure 5d). Lastly, the higher polarization loss can be expected due to non-homogeneous surface characteristics of the 1200 °C activated carbon fabric shown in Figure 5d.

## 4. Conclusions

The Kevlar fibrous waste obtained from an industry was successfully converted into activated carbon by a simultaneous process of carbonization and physical activation in the presence of atmospheric air and CO_2_ (created by charcoal), using controlled thermal treatment in a high temperature furnace. The Kevlar fibrous waste was heated under a layer of charcoal using a novel single stage carbonization and physical activation. No intermediate stabilization step was employed and direct carbonization of Kevlar fibrous waste was carried out at 800 °C, 1000 °C, and 1200 °C temperatures with the heating rate of 300 °C h^−1^ and without any holding time. 

The pyrolysis at 1200 °C resulted in activated carbon having a higher specific surface area and higher electrical conductivity. The lower heating rate was found to have a significant effect on the development of porous morphology with higher surface area. This behavior is attributed to gradual reaction of atmospheric oxygen and CO_2_ with carbonized acrylic fibrous waste. The yield of activated carbon was found to be 31% after the 1200 °C carbonization. The reduced stiffness of carbonized Kevlar was attributed to increased porosity, reduced inter-fiber/yarn friction, and abrasion at fiber/yarn cross-over points due to decomposition of organic substances during the carbonization. This limit in pore development with an increase in carbonization temperature can be explained due to gasification-induced densification effect in Kevlar fibers. The 1200 °C activated carbon sample exhibited almost 10^4^ and 10^3^ times reduction in surface and volume resistivity, respectively, over the 800 °C activated carbon sample, which is attributed to a higher graphite content, uniform distribution of graphite layers, and reduced fiber diameter in the 1200 °C activated carbon fabric. The greater number of nomadic charges (i.e., graphite content), uniform dispersion of graphite layers, reduced fiber diameter, elongated electrons’ mean free paths, larger surface area, higher porosity, and enhanced conductive network formation were responsible for the increase in multiple internal reflections and stronger absorption of EM radiations in the 1200 °C activated carbon. The use of Kevlar waste material collected from industries removes raw material procurement costs. The single-step carbonization process is less time consuming and, hence, uses relatively less power in comparison to the other procedures for carbonization through physical activation. Finally, the utilization of charcoal as the medium for heat, pyrolysis, and CO_2_ atmosphere to enhance decomposition further reduces the costs as it is cheaper, more common, and easily available. These aspects strongly contribute to the manufacturing and production costs, whilst effectively keeping in line with the primary objective of textile re-use, recycling, and waste management.

## Figures and Tables

**Table 1:** Effect of carbonization temperature on physical properties of activated carbon fabric.

Sample	Yarn Count (dtex)	Areal Density (g/m^2^)	Thickness (mm)	Shrinkage	Flexibility	Dusting	Yield (%)
Kevlar	1710	1031	1.16	-	-	-	-
800 °C	715	600.5	0.61	Good	Average	Good	58.24
1000 °C	450	368	0.54	Good	Good	Average	35.69
1200 °C	410	322.5	0.46	Average	Excellent	Poor	31.28

**Table 2:** Effect of carbonization temperature on mechanical properties of activated carbon fabric.

Sample	Stiffness (N.m)	Breaking Force (N)	Breaking Elongation (%)
Kevlar	40.92 ± 8.13	126.74 ± 9.07	1.79 ± 0.23
800 °C	8.48 ± 1.17	2.7 ± 1.30	1.15 ± 0.49
1000 °C	5.38 ± 1.01	2.25 ± 1.06	1.07 ± 0.49
1200 °C	4.16 ± 0.93	2.09 ± 1.01	0.94 ± 0.27

**Table 3:** Effect of carbonization temperature on elemental composition of activated carbon fabric.

Wt.%	C	N	O	Na	S	Cl	K	Ca
Kevlar	63.85	15.09	18.84	1.20	0.84	0.10	0.01	0.08
800 °C	69.17	8.66	13.19	4.86	2.07	0.18	0.79	1.07
1000 °C	83.47	5.68	7.81	0.45	0.69	0.02	1.68	0.20
1200 °C	89.26	2.56	5.87	0.16	0.60	0.15	1.22	0.17

**Table 4:** Effect of carbonization temperature on electrical resistivity of activated carbon fabric.

Carbonization Temperature	Surface Resistivity (ohm)	Volume Resistivity (ohm.cm)
800 °C	1.60 × 10^6^	967.14 × 10^3^
1000 °C	486.15	1251.29
1200 °C	95.78	414.14

## Data Availability

Not applicable.
